# Chronic ankle instability: a cadaveric anatomical and 3D high-resolution MRI study for surgical reconstruction procedures

**DOI:** 10.1186/s13244-024-01824-3

**Published:** 2024-10-14

**Authors:** Meng Dai, Hu Zhao, Peng Sun, Jiazheng Wang, Caixia Kong, Xiaoming Liu, Deyu Duan, Xi Liu

**Affiliations:** 1 Department of Radiology, Union Hospital, Tongji Medical College, Huazhong University of Science and Technology, 430022 Wuhan, China; 2 Hubei Province Key Laboratory of Molecular Imaging, 430022 Wuhan, China; 3 Department of Human Anatomy, School of Basic Medicine, Tongji Medical College, Huazhong University of Science and Technology, 430022 Wuhan, China; 4 National Demonstration Center for Experimental Basic Medical Education, Huazhong University of Science and Technology, 430022 Wuhan, China; 5 MSC Clinical & Technical Solutions, Philips Healthcare, 100000 Beijing, China; 6 Department of Endocrinology, Traditional Chinese and Western Medicine Hospital of Wuhan, Tongji Medical College, Huazhong University of Science and Technology, 430022 Wuhan, China; 7 Department of Orthopaedics, Union Hospital, Tongji Medical College, Huazhong University of Science and Technology, 430022 Wuhan, China

**Keywords:** Chronic ankle instability, Anterior talofibular ligament, Calcaneofibular ligament

## Abstract

**Objectives:**

To quantitatively investigate the anatomy of the anterior talofibular ligament (ATFL) and calcaneofibular ligament (CFL) for surgical reconstruction procedures in chronic ankle instability (CAI).

**Methods:**

3D MRI was performed on five fresh-frozen cadaveric ankles using six different spatial resolutions (0.3 × 0.3 × 0.3 mm^3^, 0.45 × 0.45 × 0.45 mm^3^, 0.6 × 0.6 × 0.6 mm^3^, 0.75 × 0.75 × 0.75 mm^3^, 0.9 × 0.9 × 0.9 mm^3^, 1.05 × 1.05 × 1.05 mm^3^). After comparing the MRI results with cadaver dissection, a resolution of 0.45 × 0.45 × 0.45 mm³ was selected for bilateral ankles MRI on 24 volunteers. Classification of the ATFL and four distances of surgically relevant bony landmarkers were analyzed (distance 1 and 3, the fibular origin of the ATFL and CFL to the tip of fibula, respectively; distance 2, the talar insertion of the ATFL to the bare zone of talus; distance 4, the calcaneal insertion of the CFL to the peroneal tubercle).

**Results:**

In subjective evaluation, the interobserver ICC was 0.95 (95% confidence interval (CI): 0.94–0.97) between two readers. The spatial resolution of 0.3 × 0.3 × 0.3 mm^3^ and 0.45 × 0.45 × 0.45 mm^3^ received highest subjective score on average and demonstrated highest consistency with autopsy measurements in objective evaluation. Measurements on the 48 volunteer ankles, distance 1 in type I and II were 12.65 ± 2.08 mm, 13.43 ± 2.06 mm (superior-banded in Type II) and 7.69 ± 2.56 mm (inferior-banded in Type II) (means ± SD), respectively. Distance 2 in type I and II were 10.90 ± 2.24 mm, 11.07 ± 2.66 mm (superior-banded in Type II), and 18.44 ± 3.28 mm (inferior-banded in Type II), respectively. Distance 3 and 4 were 4.71 ± 1.04 mm and 14.35 ± 2.22 mm, respectively.

**Conclusion:**

We demonstrated the feasibility of quantifying the distances between bony landmarkers for surgical reconstruction surgery in CAI using high-resolution 3D MRI.

**Critical relevance statement:**

High-resolution 3D MRI examination may have a guiding effect on the preoperative evaluation of chronic ankle instability patients.

**Key Points:**

- Spatial resolutions of 0.3 × 0.3 × 0.3 mm^3^ and 0.45 × 0.45 × 0.45 mm^3^ demonstrated highest consistency with autopsy measurements.
- The spatial resolution of 0.45 × 0.45 × 0.45 mm^3^ was conformed more to clinical needs.
- 3D MRI can assist surgeons in developing preoperative plans for chronic ankle instability.

**Graphical Abstract:**

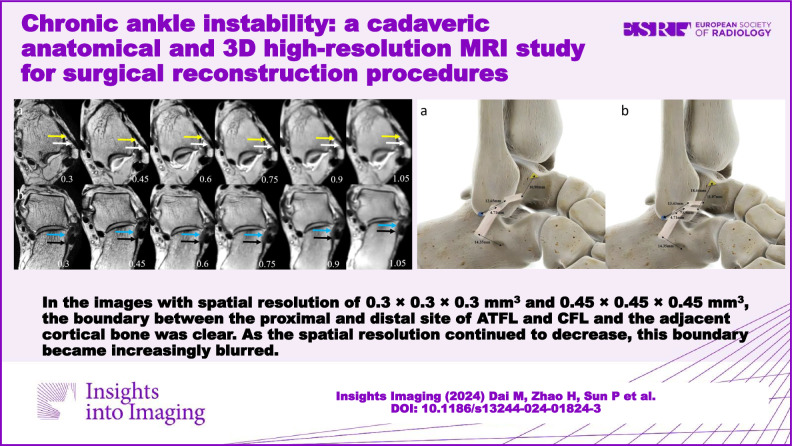

## Introduction

Ankle sprains are the most common sports-related injury. Sprains of the lateral ankle ligament complex, which consists of the anterior talofibular ligament (ATFL), posterior talofibular ligament (PTFL), and calcaneofibular ligament (CFL), are the most common type of ankle sprain [1–4]. Eighty percent of patients have ATFL tears, whereas the remaining patients have composite injuries to the ATFL and CFL [1, 2, 5]. Most lateral ankle sprains are treated nonoperatively with anti-inflammatory medication, ice, elevation, proprioceptive, and/or muscle-strengthening exercises. On the other hand, 10% to 30% of patients fail to positively response to conservative treatment and progress to chronic ankle instability (CAI), which may require surgical treatment to improve pain and function [1, 6]. Most ankle stabilization surgeries are designed to repair or reconstruct the ATFL and/or CFL, as these are the most commonly injured ankle ligaments [2, 7]. Recently, there has been an increase in minimally invasive surgery for the treatment of CAI because it reduces the incidence of postoperative complications compared to traditional open surgical procedures [7–9]. This minimally invasive surgery (MIS) for CAI includes anatomical repair and reconstruction of the ATFL and/or CFL. The MIS requires the use of bone anchors, a surgical instrument, or the construction of bone tunnels at the anatomical origins and insertions of the ATFL and/or CFL [7]. Therefore, it is important to evaluate the anatomical origins and insertions of the ATFL and CFL quantitatively to facilitate the preoperative plan of minimally invasive surgeries. Although previous studies have focused on the anatomical origins and insertions of the ATFL and CFL using embalmed specimens or fresh-frozen specimens [10–14], similar studies are rarely seen on living subjects using non-invasive magnetic resonance imaging (MRI).

Three-dimensional (3D) MRI with high spatial resolution, especially 3D isotropic MRI, has been widely used to obtain thin-section images and and multiplanar reformation images that might help evaluate thin ligament structures [15–18]. For instance, Teramoto et al evaluated the morphological characteristics of the normal lateral ankle ligament in injured patients and uninjured controls [19]; Choo et al [20] studied the calculation of the number of bands in ATFL and evaluated the normal MRI characteristics of each band of the ATFL in asymptomatic volunteers.

In this study, we aimed to investigate the feasibility of quantifying the distances of surgically relevant bony landmarkers for repair and reconstructive surgery of the lateral ankle ligaments using high-resolution 3D MRI. An arthroscopic procedure that can easily locate the bone anchors for constructing bone tunnels in individuals in vivo for preoperative planning was hypothesized.

## Materials and methods

This study was approved by the Ethics Committee of our hospital (No. 0456-01).

### Frozen cadaveric specimens

A total of five fresh-frozen cadaveric ankles (four left and one right ankles) from five different donors (men: women, 3:2, mean age of 71.2 years, range from 60 to 79 years) were involved in the study, with an average foot length of 21.4 mm (range from 17.7 mm to 23.8 mm). All donors had no evidence of prior external foot injuries or surgical procedures on the ankle.

### MRI on specimens

The specimens were examined using a 3.0-T magnetic resonance system (Ingenia CX, Philips Healthcare, Best, the Netherlands) with an eight-channel phased-array ankle coil (dS FootAnkle 8ch 3.0 T, Philips Healthcare). All fresh-frozen cadaveric ankles were placed in a neutral position and scanned using a sagittal 3D proton density (PD)-weighted Volumetric ISotropic variable flip angle Turbo-spin-echo Acquisition sequence (VISTA, Philips Healthcare) without fat suppression. All specimens underwent six MRI scans with different spatial resolutions to determine the optimal resolution for quantifying the distance from origin and insertion to bony landmarks. The details of the MR protocol are described in Table 1.

**Table 1:** MR sequence parameters

	0.3 × 0.3 × 0.3 mm^3^	0.45 × 0.45 × 0.45 mm^3^	0.6 × 0.6 × 0.6 mm^3^	0.75 × 0.75 × 0.75 mm^3^	0.9 × 0.9 × 0.9 mm^3^	1.05 × 1.05 × 1.05 mm^3^
Repetition time/time to echo (ms)	1000/45	1000/45	1000/45	1000/45	1000/45	1000/45
Field of view (mm^3^)	180 × 160 × 180	180 × 160 × 180	180 × 160 × 180	180 × 160 × 180	180 × 160 × 180	180 × 160 × 180
Acquisition voxel size	0.3 × 0.3 × 0.3 mm^3^	0.45 × 0.45 × 0.45 mm^3^	0.6 × 0.6 × 0.6 mm^3^	0.75 × 0.75 × 0.75 mm^3^	0.9 × 0.9 × 0.9 mm^3^	1.05 × 1.05 × 1.05 mm^3^
Echo-train length	63	63	63	63	63	63
Compressed sensing acceleration factor	6	6	6	6	6	6
Flip angle	Variable flip angle	Variable flip angle	Variable flip angle	Variable flip angle	Variable flip angle	Variable flip angle
Refocusing radiofrequency pulse	65	65	65	65	65	65
Scan time	14 min 13 s	5 min 56 s	3 min 21 s	2 min 8 s	1 min 30 s	1 min 6 s

### Image and autopsy analysis

A total of 9555 images of five fresh-frozen specimens from all six protocols were obtained and visually checked to ensure sufficient image quality for analysis. Two radiologists (X.L. and M.D.) with 22 and 10 years of experience in foot and ankle radiology, respectively, evaluated the images (provided in a random order), blinded to the sequence information. Any disagreement was solved by discussion and consensus between the two radiologists. ATFL and CFL subjective scores, morphologic parameters, and the distances from bony landmarks to the centers of the footprint of ATFL and CFL were noted.

Subjective scoring for the ATFL and CFL followed a four-point scale (0, poor; 1, fair; 2, good; 3, excellent) [17, 21] on the following aspects: (1) whether the reader could completely resolve from the fibular origin to talar insertion of ATFL and from the fibular origin to calcaneal insertion of the CFL; (2) whether the reader could resolve the ATFL and CFL from medial margin to lateral margin; (3) whether the reader could resolve the ATFL and CFL from upper margin to lower margin; and (4) the image sharpness that distinguish ATFL and CFL from the surrounding soft tissues.

**Fig. 1:**
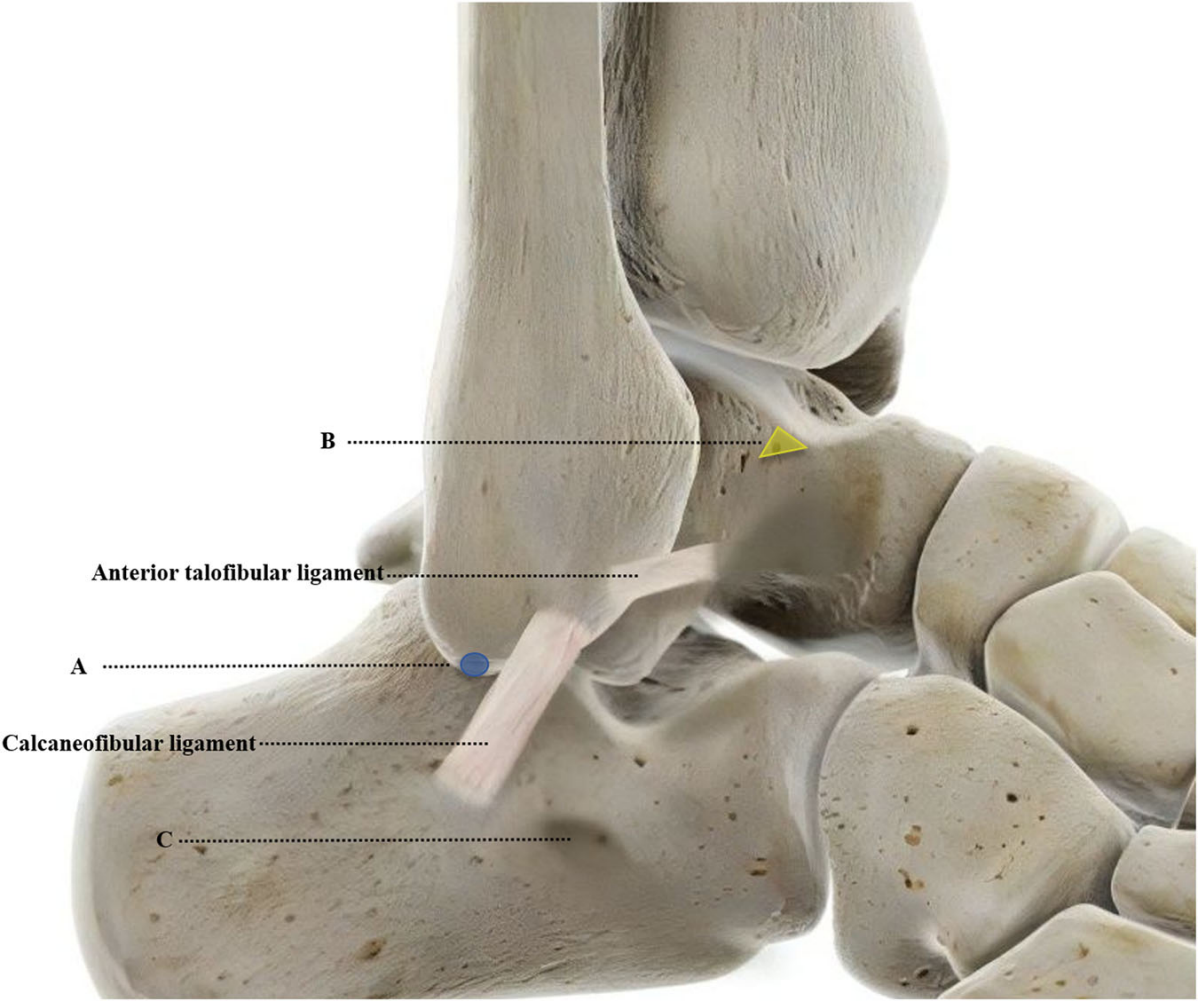
Lateral view of a right ankle depicting the anatomic attachment sites of the anterior talofibular ligament and calcaneofibular ligament and the reference bony landmarks used for the measurement. A: Tip of the fibula (blue circle). B: Bare zone of the talus (yellow triangle). C: Peroneal tubercle

Morphologic parameters of the ATFL and CFL include fiber bundle length and distance from bony landmarks to the center of the footprint of ATFL and CFL. The length was measured in the central portion of the ATFL and CFL from the origin to the insertion. The bony landmarks identified for this study were defined as follows (Fig. 1):

1. The tip of the fibula (TF) was defined as distal and posterior to the articular tip of the fibula [11].
2. The bare zone of the talus (BZT) was defined as an area consistently present behind the talar neck, in front of the lateral articular surface of the talus, below the anterior cartilaginous part of the talar dome, and above the distal ATFL insertion [22].
3. Peroneal tubercle (PT) was defined as a structure projected from the anterior third of the lateral border of the calcaneus [11].

Distance from bony landmarks to the center of the footprint of ATFL and CFL includes four distances: distance 1, from the fibular origin of the ATFL to the TF; distance 2, from the talar insertion of the ATFL to the BZT; distance 3, from the fibular origin of the CFL to the TF; distance 4, from the calcaneal insertion of the CFL to the PT.

Finally, a cadaver dissection was performed to validate the MRI data. The morphologic parameters and the distance from bony landmarks to the center of the previously defined footprint of ATFL and CFL were again measured.

### MRI on volunteers

Twenty-four healthy adult volunteers, eleven males and thirteen females (mean age 27.2 years, age range, 13–40 years) with a mean body mass index (BMI) of 22.38 (range 17.22–31.28), were recruited for this prospective study. The healthy volunteers presented no history of the sprain to either ankle and no previous surgical operations to the musculoskeletal structures in either limb of the lower extremity. All healthy adult volunteers were examined on both two ankles using the aforementioned MR scanner. Resolution of 0.45 × 0.45 × 0.45 mm^3^ was performed for the 3D isotropic imaging based on the evaluation results of the cadaver study. The resulting images were transferred to the post-processing workstation for analysis of morphologic parameters, including the classification of ATFL (by the number of ATFL fiber bundles using criteria described by Edama et al [23], which means Type I has one fiber bundle and Type II has two fiber bundles—including superior-banded and inferior-banded), the length of ATFL and CFL, and the determination of the four previously defined distances from bony landmarks to the center of the footprint of ATFL and CFL.

### Statistical analysis

The interobserver consistency of subjective scores and objective measurements was evaluated by two-way random model intraclass correlation coefficients (ICCs) and the Bland-Altman plot. The Spearman Rank Correlation was used to investigate the correlation between the subjective scoring and different MRI protocols. The results of measurements from autopsy and MRI were displayed using a violin plot. The Friedman rank-sum test was utilized to evaluate the measurement differences (systematic bias) between autopsy and MRI with different resolutions. A post hoc analysis was conducted to test pairwise differences and account for the multiple comparison correction. Two-way mixed model ICCs and Bland-Altman plot were calculated to assess the pairwise measurement agreement between autopsy and different MRI protocols with different spatial resolutions. A *p* < 0.05 was considered to be statistically significant. Consistency based on ICCs was classified using the following criteria: 0–0.39, poor; 0.40–0.59, fair; 0.60–0.74, good; and 0.75–1.0, excellent [24].

## Results

### Subjective evaluation

**Fig. 2:**
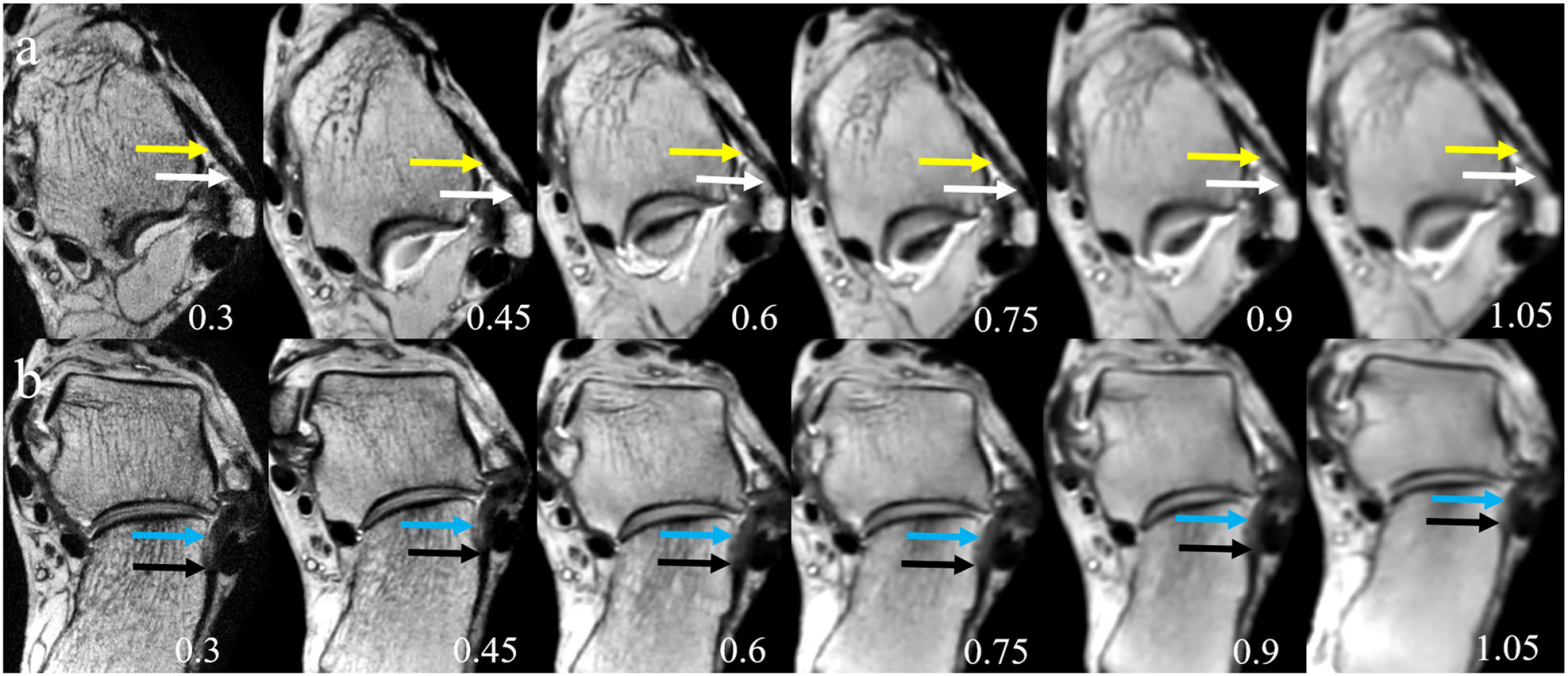
Three-dimensional magnetic resonance imaging reconstruction, showing the entire length of anterior talofibular ligament (ATFL) (**a**, axial plane, yellow arrow), calcaneofibular ligament (CFL) (**b**, oblique coronal plane, blue arrow), the proximal site of ATFL (**a**, white arrow), and the distal site of CFL (**b**, black arrow) in all six spatial resolutions. In the spatial resolution of 0.3 × 0.3 × 0.3 mm^3^ and 0.45 × 0.45 × 0.45 mm^3^, the boundary between the ligament and the adjacent cortical bone in the proximal or distal site was clear. In the spatial resolution of 0.6 × 0.6 × 0.6 mm^3^ and 0.75 × 0.75 × 0.75 mm^3^, the boundary between the ligament and cortical bone becomes increasingly blurred. In spatial resolutions of 0.9 × 0.9 × 0.9 mm^3^ and 1.05 × 1.05 × 1.05 mm^3^, the boundary between the ligament and cortical bone was completely indistinguishable. At lower resolution, it was difficult to measure the entire length of ATFL and CFL (from the fibular origin to talar insertion of ATFL, fibular origin to calcaneal insertion of the CFL) and the entire thickness (from medial margin to lateral margin of the ATFL and CFL)

The results of subjective scoring for anatomical ligament identification were summarized in Supplementary Table 1. The ICC values of the two readers were 0.95 (95% confidence interval (CI): 0.94–0.97). Using Spearman Rank Correlation, the correlation coefficient between the subjective scoring and MRI resolution was −0.82. The images with spatial resolution of 0.3 × 0.3 × 0.3 mm^3^ and 0.45 × 0.45 × 0.45 mm^3^ had the highest percentage of score 3 (Supplementary Fig. S1). Figure 2 shows representative MR images at different spatial resolutions demonstrating the entire length of ATFL and CFL, the proximal site of ATFL, and the distal site of CFL labeled. In the images with spatial resolution of 0.3 × 0.3 × 0.3 mm^3^ and 0.45 × 0.45 × 0.45 mm^3^, the boundary between the proximal and distal site of ATFL and CFL and the adjacent cortical bone was clear, and as the spatial resolution continued to decrease, this boundary became increasingly blurred. The boundary was completely indistinguishable in images with spatial resolutions of 0.9 × 0.9 × 0.9 mm^3^ and 1.05 × 1.05 × 1.05 mm^3^.

### Objective evaluation

**Fig. 3:**
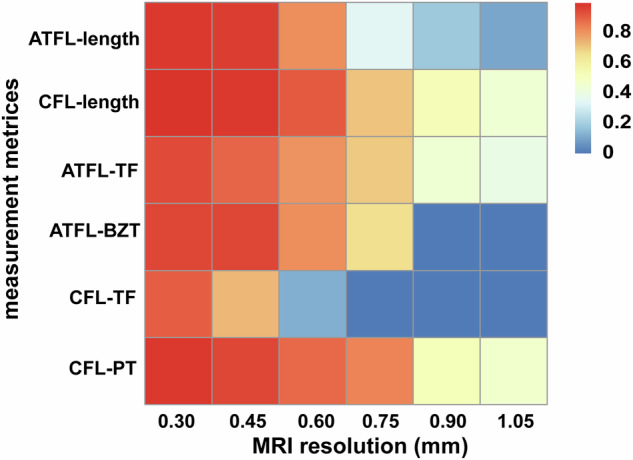
Intraclass correlation coefficients between measurements from the autopsy data and MRI with different spatial resolutions. ATFL, anterior talofibular ligament; CFL, calcaneofibular ligament; ATFL-TF, distance between ATFL and the tip of the fibula; ATFL-BZT, distance between ATFL and bare zone of the talus; CFL-TF, distance between CFL and tip of the fibula; CFL-PT, distance between CFL and peroneal tubercle

Figure 3 shows the ICCs between measurements from the autopsy data and MRI with different spatial resolutions. For all morphologic parameters and the distance from bony landmarks to the center of the footprint of ATFL and CFL, the highest consistency was obtained at a spatial resolution of 0.3 × 0.3 × 0.3 mm^3^ and 0.45 × 0.45 × 0.45 mm^3^ (except for distance 3), displayed as a distinct dark red in the ICC heat map (Fig. 3).

**Fig. 4:**
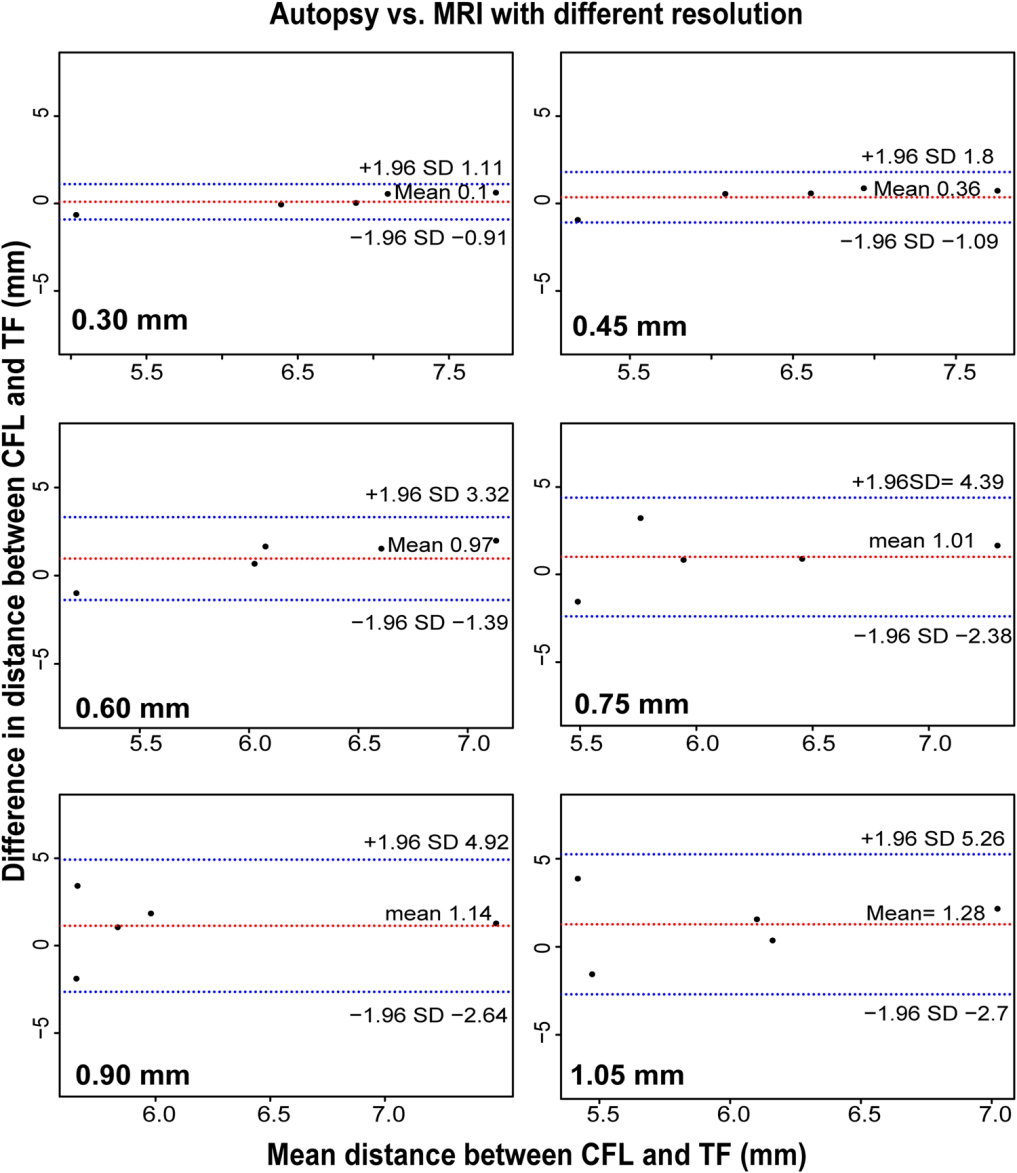
Bland-Altman plots of the measurement consistency between the autopsy and MRI with different spatial resolutions. Differences in the distance between calcaneofibular ligament (CFL) and the tip of the fibula (TF) (*y*-axis) were plotted against the mean distance between CFL and TF (*x*-axis). The red dashed line indicates the mean difference. Top and bottom blue dashed lines correspond to upper and lower margins of 95% limits of agreement. With a probability of 95%, differences in distance measured by autopsy and MRI between CFL and TF of future examinations will be between the upper and lower limits of agreement (mean ± variability estimate = 1.96 standard deviation (SD))

**Fig. 5:**
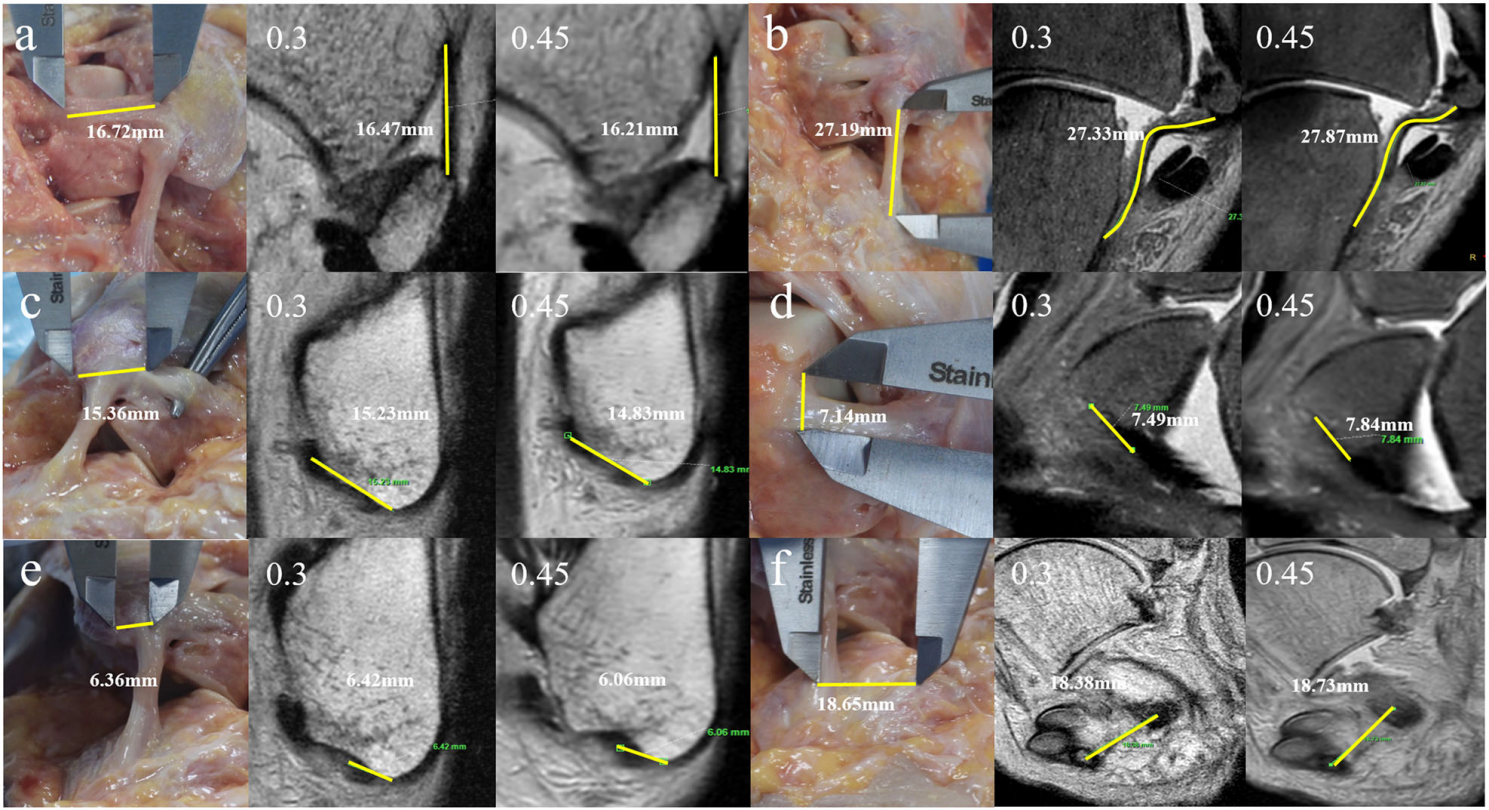
Measurements from the autopsy data and MRI in spatial resolutions of 0.3 × 0.3 × 0.3 mm^3^ and 0.45 × 0.45 × 0.45 mm^3^. **a** The entire length of anterior talofibular ligament (ATFL). **b** The entire length of calcaneofibular ligament (CFL). **c** From the fibular origin of the ATFL to the tip of the fibula. **d** From the talar insertion of the ATFL to the bare zone of the talus. **e** From the fibular origin of the CFL to the tip of the fibula. **f** From the calcaneal insertion of the CFL to the peroneal tubercle

The Bland-Altman plots, which indicate the measurement consistency of different distances (distances 1–4) between the autopsy and MRI with different spatial resolutions, are shown in Fig. 4 and Supplementary Figs. S2–4. Regarding the distance from bony landmarks to the center of the footprint of ATFL and CFL, the measurement consistency between the autopsy and MRI was improved at higher resolution, while the highest consistency obtained at spatial resolutions of 0.3 × 0.3 × 0.3 mm^3^ (mean differences and 95% LOAs: −0.31 and −1.32 to 0.69 (Distance 1), −0.03 and −1.16 to 1.09 (Distance 2), 0.1 and −0.91 to 1.11 (Distance 3), −0.27 and −1.33 to 0.79 (Distance 4)) and 0.45 × 0.45 × 0.45 mm^3^ (mean differences and 95% LOA: −0.32 and −1.81 to 1.18 (Distance 1), −0.05 and −1.16 to 1.06 (Distance 2), 0.36 and −1.09 to 1.8 (Distance 3), 0.86 and −1.68 to −0.03 (Distance 4)) (Figs. 4, 5 and Supplementary Figs. S2–4). The violin plots of measurements of the autopsy and MRI with different spatial resolutions are presented in Supplementary Fig. S5.

### Results for the volunteers

**Fig. 6:**
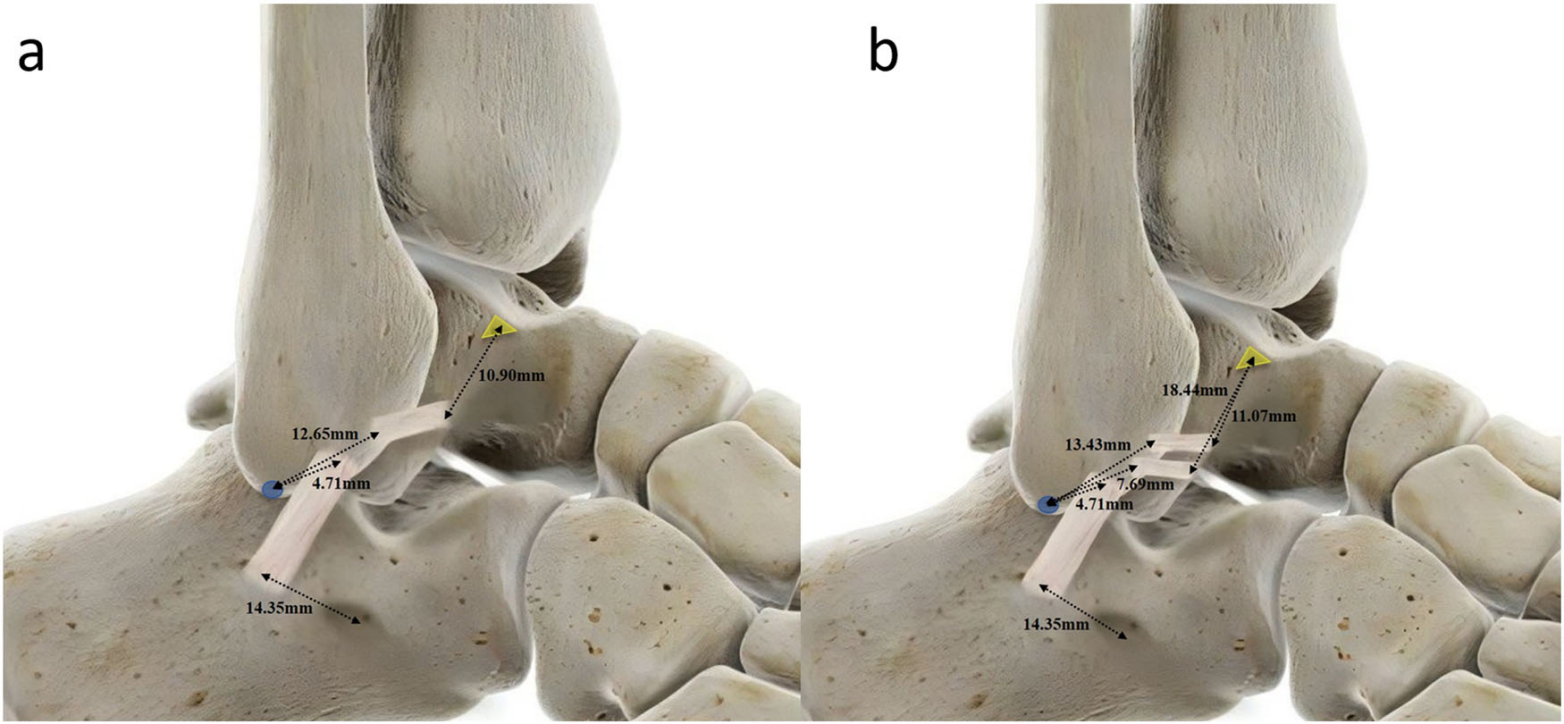
Lateral view of a right ankle depicting the measurement results for the volunteers. **a** Distances from bony landmarks to the calcaneofibular ligament and single-banded anterior talofibular ligament. **b** Distances from bony landmarks to the calcaneofibular ligament and the double-banded anterior talofibular ligament

A total of 48 ankles were evaluated in our study. Among the 48 ankles data, 22 (45.8%) and 26 (54.2%) ankles were classified into Types I and II of ATFL, respectively. Table 2 and Fig. 6 show the distance from bony landmarks to the center of the footprint of ATFL and CFL.

**Table 2:** Distance from bony landmarks to origin and insertion of the ATFL and CFL in 48 ankles (mm)

		Distance, means ± SD
ATFL		
Type I	From the fibular origin of the ATFL to the tip of the fibula	12.65 ± 2.08
	From the talar insertion of the ATFL to the bare zone of the talus	10.90 ± 2.24
Type II		
Superior-banded	From the fibular origin of the ATFL to the tip of the fibula	13.43 ± 2.06
	From the talar insertion of the ATFL to the bare zone of the talus	11.07 ± 2.66
Inferior-banded	From the fibular origin of the ATFL to the tip of the fibula	7.69 ± 2.56
	From the talar insertion of the ATFL to the bare zone of the talus	18.44 ± 3.28
CFL	From the fibular origin of the CFL to the tip of the fibula	4.71 ± 1.04
	From the calcaneal insertion of the CFL to the peroneal tubercle	14.35 ± 2.22

*ATFL* anterior talofibular ligament, *CFL* calcaneofibular ligament

## Discussion

The most important observation of the present study was that the spatial resolution of 0.45 × 0.45 × 0.45 mm^3^ or higher is sufficient for measuring the distance from bony landmarks to anatomical origins and insertions of the ATFL and CFL and morphologic parameters. In addition, our study on the volunteers showed that the origin of the single fiber bundle, superior fiber bundle, inferior fiber bundle of the ATFL and CFL were located around 12.65 mm, 13.43 mm, 7.69 mm, and 4.71 mm from the tip of the fibula, respectively. The bare zone of the talus was located about 10.90 mm, 11.07 mm, 18.44 mm to the single fiber bundle, superior fiber bundle, inferior fiber bundle of the ATFL insert, respectively. The peroneal tubercle was located about 14.35 mm to the CFL insert.

Several studies [16, 18, 20, 25–27] have confirmed the adequate image quality of 3D isotropic acquisition in the ankle joints and the superior detectability for the ankle, where the suggested spatial resolution and acquisition time were 0.6 × 0.6 × 0.6 mm^3^ and 6:43 min [25], 0.5 × 0.5 × 0.5 mm^3^ and 7:31 min [26], 0.5 × 0.5 × 0.5 mm^3^ and 4:46 min [27], and 0.5 × 0.5 × 0.5 mm^3^ and 5:20 min [18]. However, the optimal spatial resolution for the standard image quality of lateral ankle ligament has not been adequately studied. In our study, we found that a resolution of 0.45 × 0.45 × 0.45 mm^3^ or higher could help accurately analyze the length of the ligaments and the distance from bony landmarks to anatomical origins and insertions of the ATFL and CFL. However, one exception was that the ICC of distance 3 (from the fibular footprint centers of the CFL to the TF) between the autopsy data and MRI with a spatial resolution of 0.45 × 0.45 × 0.45 mm^3^ was just 0.74 and in spatial resolution of 0.3 × 0.3 × 0.3 mm^3^ the ICC was 0.90. This may be due to the CFL and the inferior ATFL fascicle sharing a common fibular insertion, which is interconnected by arciform fibers, forming the lateral tibiotalocalcaneal ligament complex [5, 28], thus requiring higher resolution to accurately locate the fibular footprint centers of the CFL. In addition, the fibular origin of the CFL originates just below the origin of the inferior band of the ATFL [7, 14, 28] and forms an angle of 10–50° with the long axis of the fibula [29], which caused the proximal site of the CFL to fit closely to the distal fibula. Therefore, the identification of the origin of the CFL was prone to deviation.

A main concern of high-resolution 3D MRI is the prolonged scan time. In our study, the acquisition time for 0.3 × 0.3 × 0.3 mm^3^ voxel size was 14:25, and for 0.45 × 0.45 × 0.45 mm^3^ resolution, the scan time was 05:58, both with six-fold acceleration using compressed sensing. However, it was difficult for patients to remain motionless for 14 min when undergoing MRI scans, especially when the patient’s ankle was injured and painful. Our study, therefore, suggested that the spatial resolution of 0.45 × 0.45 × 0.45 mm^3^ was more in line with clinical needs.

MIS techniques have become increasingly more common in recent years with the desired goal of reducing postoperative pain and recovery time. MIS techniques, which include anatomical repairs and reconstruction using arthroscopy [1, 7, 30], require a clearer understanding of the anatomical origin and insert of ATFL and CFL to assure precise repair or reconstruction. The previous cadaveric study described the tip of the fibula as the bony landmarks of the fibular origin of the ATFL and CFL [11]. Taser et al [14] and Wenny et al [31] suggested the anterior tubercle of the fibula as a reference point. Matsui et al [7] showed that the fibular origins of the ATFL and CFL were located around 10–14 and 5–8 mm from the tip of the fibula, respectively, in their systematic review. In our study, the origin of the single fiber bundle and the superior fiber bundle in type II of the ATFL was similar to the previous literature data. We also measured the distance of the inferior fiber bundle from the tip of the fibula in our study. The distance of the CFL from the tip of the fibula was shorter than the previous literature data. These differences in values were not significant and may be accounted for by the cohort size and race.

The subtalar joint [32], superior surface of the talar body [11, 33], the apex of the lateral talar process [33], the anterior external cartilage surface of the talus [10] were used as the bony landmarks of the talar insertion of the ATFL. However, in our study, the bare zone of the talus [22] was used as the reference bony landmarks because it is an area without cartilage coverage. Moreover, the bare zone of the talus is present above the distal ATFL insertion and can be easily detected by MRI. Our study showed that single fiber bundle, superior fiber bundle, inferior fiber bundle of the ATFL insertion of ATFL located around 10.90 mm, 11.07 mm, 18.44 mm below the bare zone of the talus.

The peroneal tubercle [11], superior surface of the calcaneus [14, 31], subtalar joint [32] were used as the bony landmarks of the calcaneal insertion of the CFL. As discussed in a previous study [11], identifying a pertinent bony landmark reference for the CFL attachment on the calcaneus is challenging, and there was no consensus on a reproducible and consistent band landmark. Matsui et al [7] showed peroneal tubercle located about 15 mm anteroinferior to the CFL insertion in their review. Our study was 14.35 mm, which was similar to the data of Matsui et al’s study. We measured the distance using the center point of the peroneal tubercle, which was easier to find in the MRI. We think that the center point of the peroneal tubercle is the most reproducible bony landmark for preoperative planning purposes because it was easily palpated during lateral ankle surgical exposures.

The present study suggests reference distances that can help surgeons locate the bone anchors or construct bone tunnels for a more anatomically accurate reconstruction of the lateral ankle ligament. As an example, we find that the single fiber bundle of the ATFL originates an average of 12.65 mm from the tip of the fibula. Although it is not surgically feasible to achieve such small measured parameters, this information will help surgeons place the graft tissue approximately 12–13 mm from the tip of the fibula, particularly in a patient who is much smaller or larger than average. The individualized measurement of the reference distances suggested in this study might be particularly important in preoperative planning.

This study has limitations. The sample size of the frozen fresh specimens was small, so we were unable to perform a more sophisticated statistical analysis on these surgical related four distances in only five specimens. Additionally, cadaveric subjects had a mean age of 71.2 years old, while the mean age range of the healthy volunteers was 27.2 years old. Although the four distances in our study were from the locations of origins or insertions to the bony landmarks, which means that these bony landmarks are relatively constant and would not undergo significant changes with age, age may still be a confounding factor. More cadaveric subjects need to be included in the study.

In conclusion, we have demonstrated the feasibility of using high-resolution 3D MRI in vivo to quantify the distances of surgically relevant bony landmarkers for repair and reconstructive surgery of the lateral ankle ligaments. A spatial resolution of higher than 0.45 × 0.45 × 0.45 mm^3^ is necessary and generally sufficient for image-based preoperative planning for the treatment of CAI. Further studies will be necessary to establish evidence regarding the clinical utility of this strategy to improve the treatment of CAI.

## Supplementary information

ELECTRONIC SUPPLEMENTARY MATERIAL

## Data Availability

The data that support the findings of this study are available on request from the corresponding author (X.L.).

## Abbreviations

ATFL: Anterior talofibular ligament
BZT: The bare zone of the talus
CAI: Chronic ankle instability
CFL: Calcaneofibular ligament
MIS: Minimally invasive surgery
PT: Peroneal tubercle
TF: The tip of the fibula
