# Supplementary material for: Chronic ankle instability: a cadaveric anatomical and 3D high-resolution MRI study for surgical reconstruction procedures
Source: Insights Imaging. 2024 Oct 14;15:249. doi: 10.1186/s13244-024-01824-3 (PMC11479647; doi:10.1186/s13244-024-01824-3)
Supplement: Supplementary file 1 — ELECTRONIC SUPPLEMENTARY MATERIAL [file 13244_2024_1824_MOESM1_ESM.pdf]

Chronic Ankle Instability: A Cadaveric Anatomical and 3D High-Resolution MRI Study for Surgical Reconstruction Procedures

ELECTRONIC SUPPLEMENTARY MATERIAL

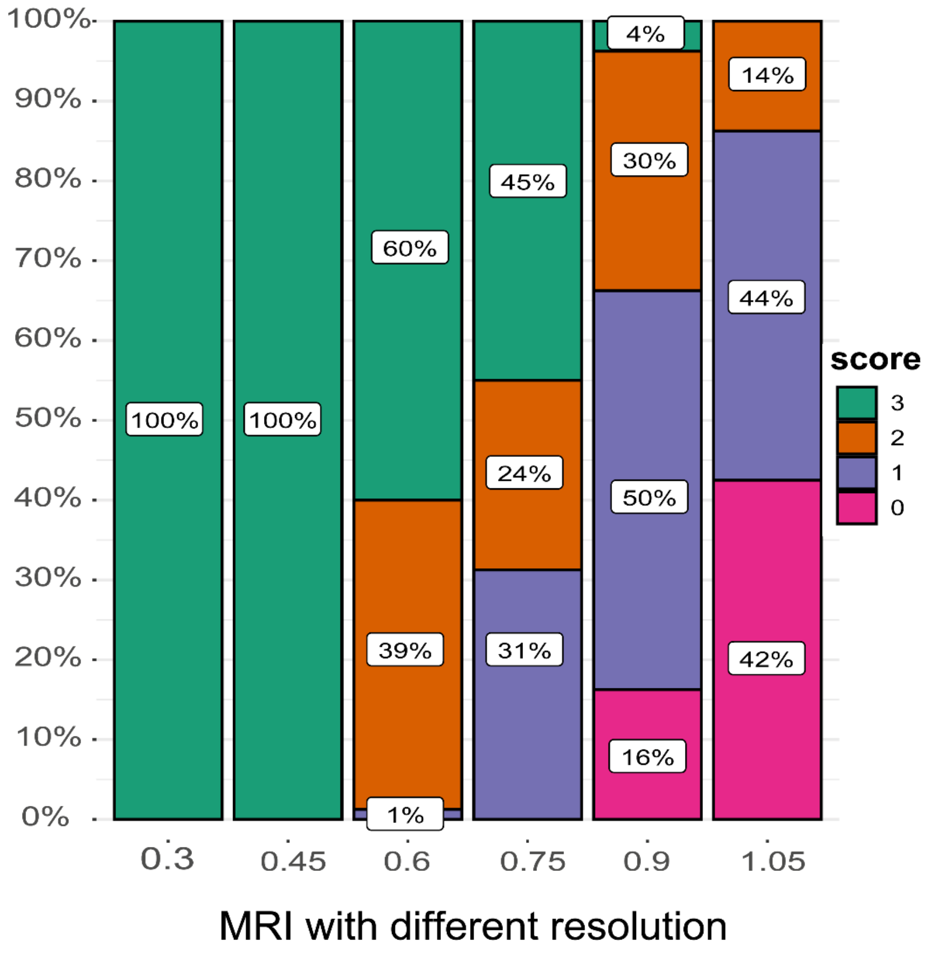

Figure S1. Percentage of the frequency of each score (0,1,2,3) in different spatial resolutions.

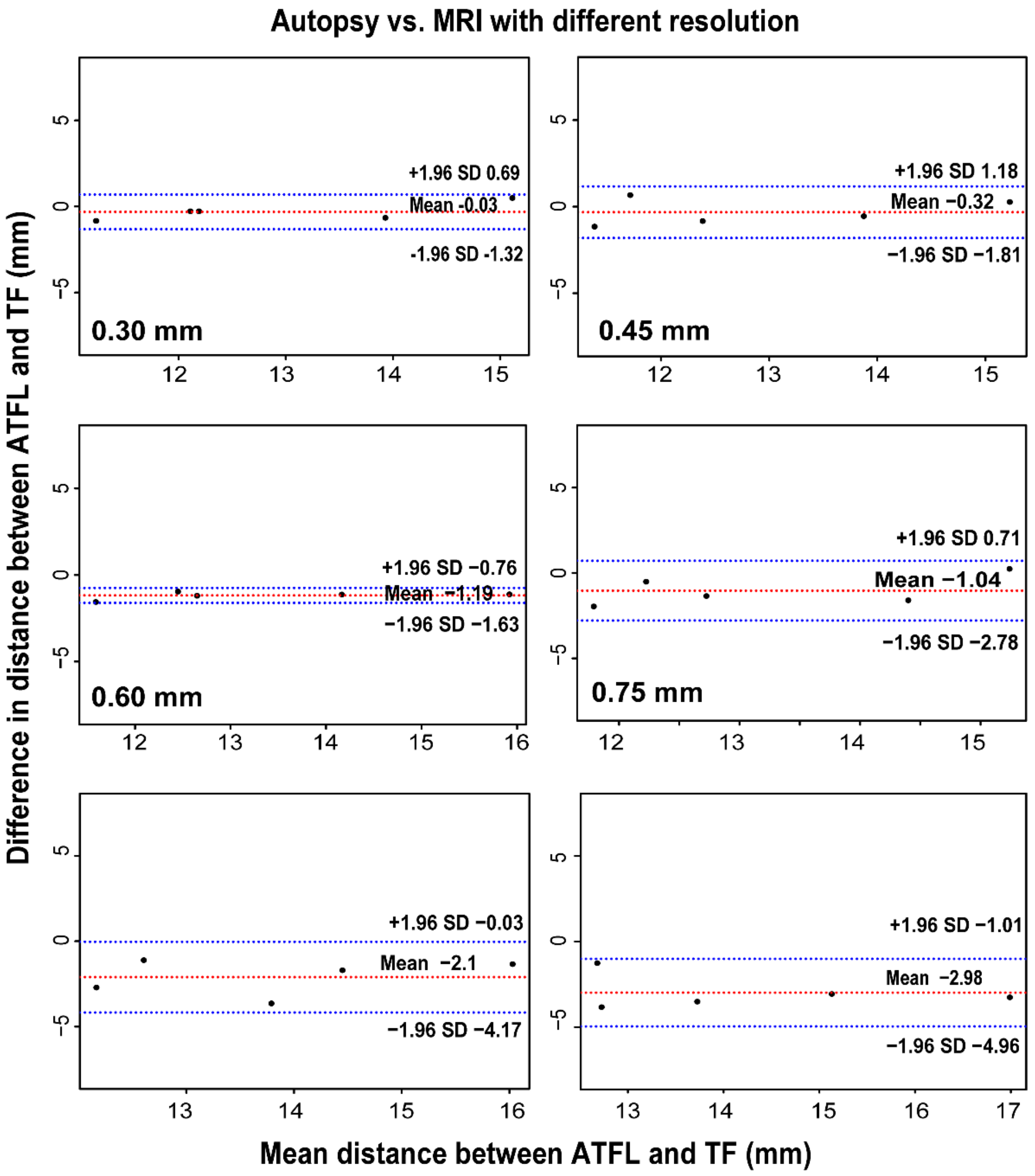

Figure S2. Bland-Altman plots of the measurement consistency between the autopsy and MRI with different spatial resolutions. Differences in distance between anterior talofibular ligament (ATFL) and the tip of the fibula(TF) (y-axis) were plotted against mean distance between ATFL and TF (x-axis). The red dashed line indicates mean difference. Top and bottom blue dashed lines correspond to upper and lower margins of 95% limits of agreement. With probability of 95%, differences in distance between ATFL and TF of future examinations will be between upper and lower limits of agreement (mean ± variability estimate = 1.96 standard deviation [SD]).

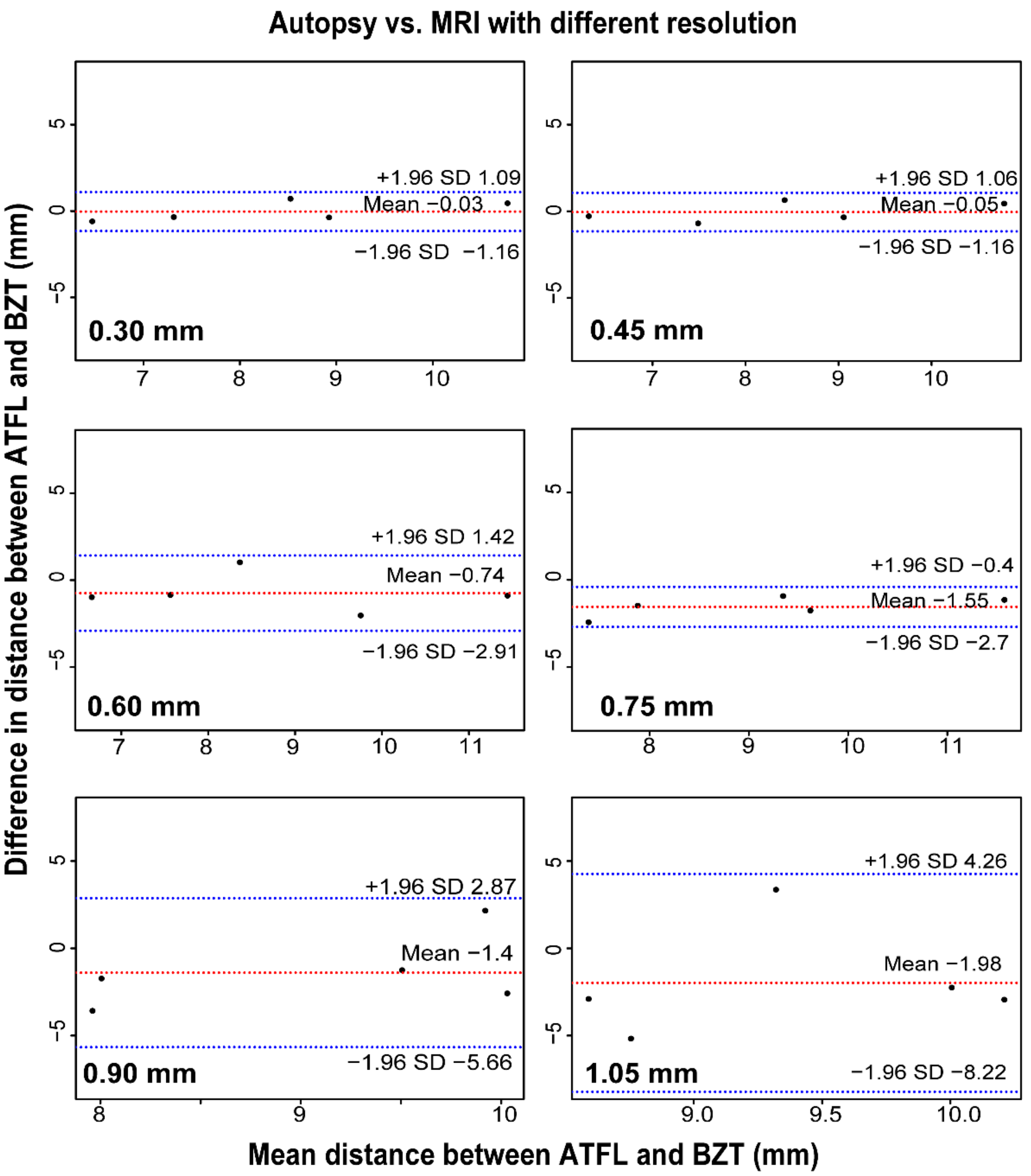

Figure S3. Bland-Altman plots of the measurement consistency between the autopsy and MRI with different spatial resolutions. Differences in distance between anterior talofibular ligament (ATFL) and bare zone of the talus (BZT) (y-axis) were plotted against mean distance between ATFL and BZT (x-axis). The red dashed line indicates mean difference. Top and bottom blue dashed lines correspond to upper and lower margins of 95% limits of agreement. With probability of 95%, differences in distance between ATFL and BZT of future examinations will be between upper and lower limits of agreement (mean ± variability estimate = 1.96 standard deviation [SD]).

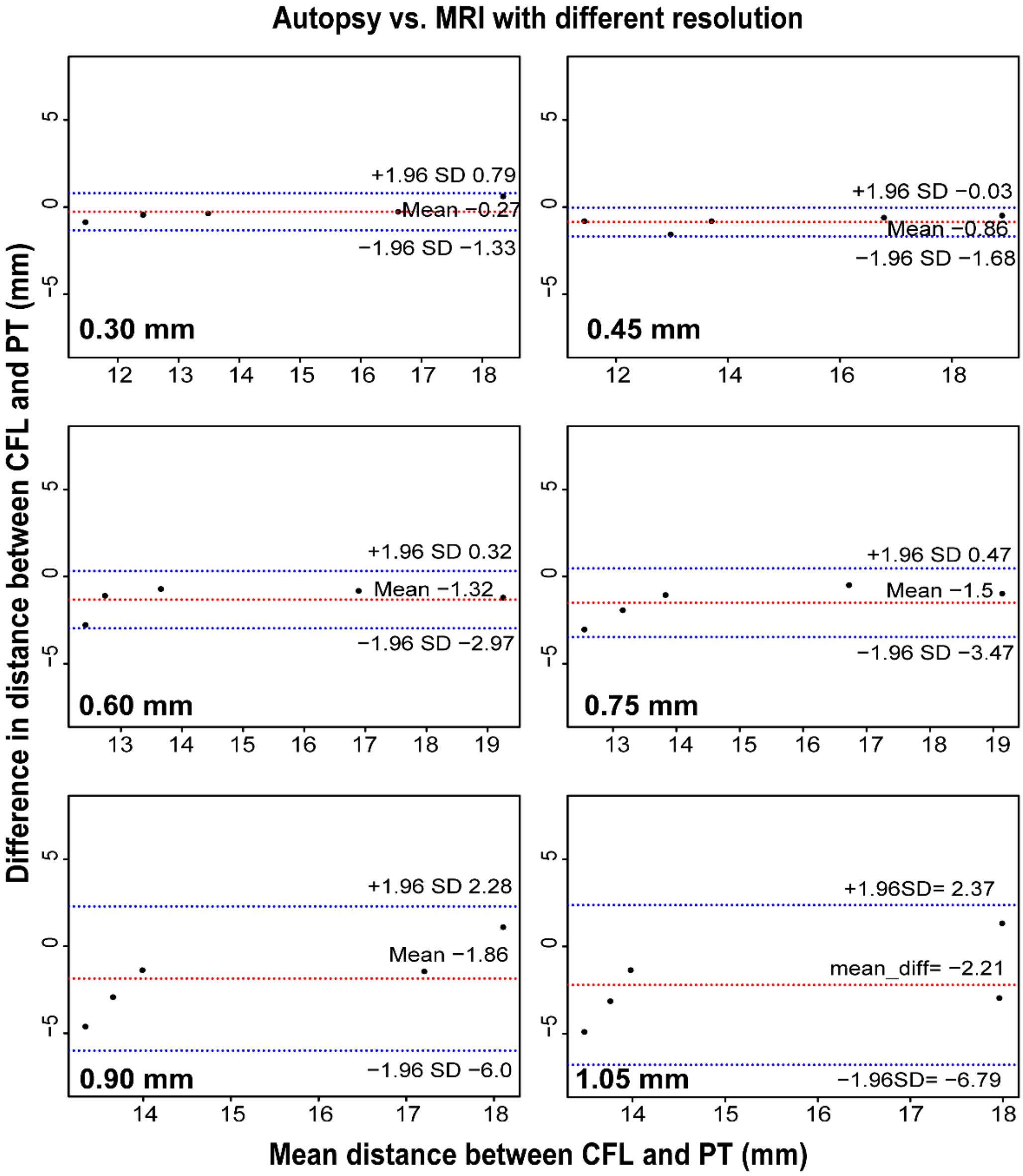

Figure S4. Bland-Altman plots of the measurement consistency between the autopsy and MRI with different spatial resolutions. Differences in distance between calcaneofibular ligament(CFL) and peroneal tubercle (PT) (y-axis) were plotted against mean distance between CFL and PT (x-axis). The red dashed line indicates mean difference. Top and bottom blue dashed lines correspond to upper and lower margins of 95% limits of agreement. With probability of 95%, differences in distance between CFL and PT of future examinations will be between upper and lower limits of agreement (mean ± variability estimate = 1.96 standard deviation [SD]).

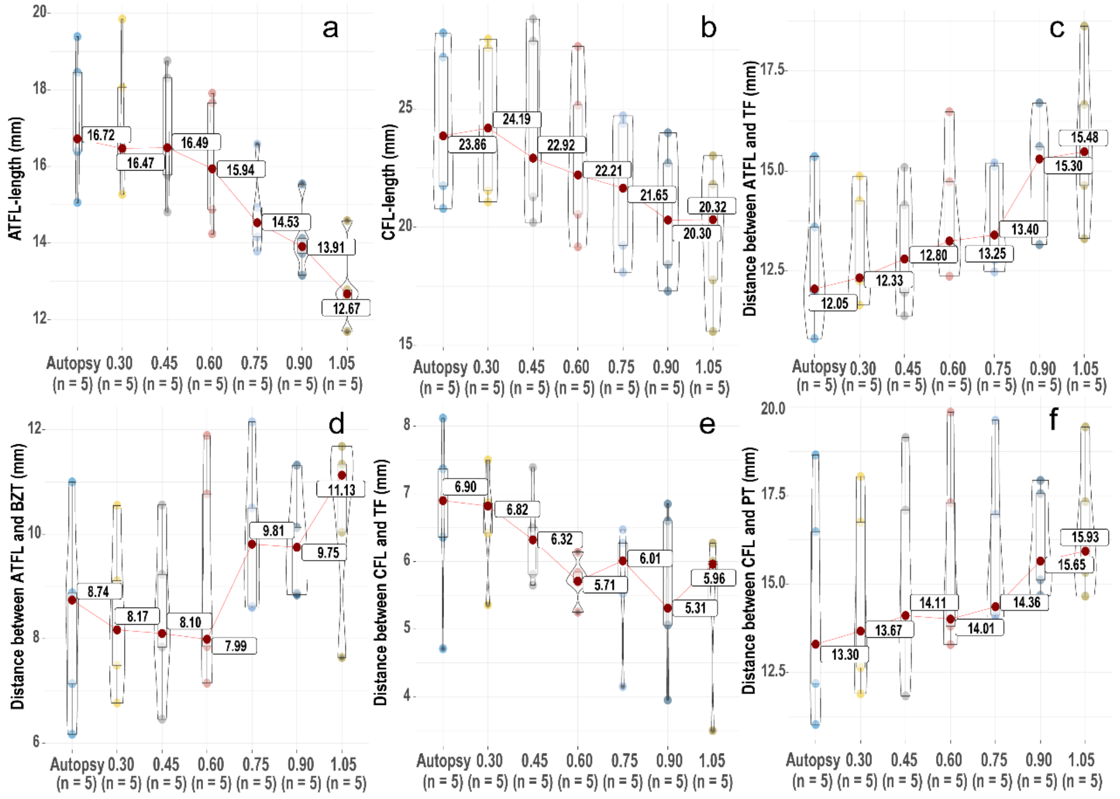

Figure S5. Violin plots of segmentation results from autopsy and MRI with different spatial resolutions for (a) the anterior talofibular ligament (ATFL) length, (b) calcaneofibular ligament(CFL) length, (c) distance between ATFL and the tip of the fibula(TF),(d) distance between ATFL and bare zone of the talus(BZT),(e) distance between CFL and TF,(f) distance between CFL and peroneal tubercle(PT).

Table S1 The frequencies of subjective scoring (0, 1, 2, 3) for the ATFL and CFL identification

score / resolutions	reader 1				reader 2			
	0	1	2	3	0	1	2	3
0.3×0.3×0.3 mm^3	0	0	0	40	0	0	0	40
0.45×0.45×0.45mm^3	0	0	0	40	0	0	0	40
0.6×0.6×0.6mm^3	0	0	16	24	0	1	15	24
0.75×0.75×0.75mm^3	0	12	10	18	0	13	9	18
0.9×0.9×0.9mm^3	5	21	12	2	8	19	12	1
1.05×1.05×1.05mm^3	14	20	6	0	20	15	5	0

Abbreviation: ATFL, anterior talofibular ligament; CFL, calcaneofibular ligament.
